# Percutaneous Hepatic Perfusion with Melphalan in Patients with Unresectable Ocular Melanoma Metastases Confined to the Liver: A Prospective Phase II Study

**DOI:** 10.1245/s10434-020-08741-x

**Published:** 2020-08-05

**Authors:** T. Susanna Meijer, Mark C. Burgmans, Eleonora M. de Leede, Lioe-Fee de Geus-Oei, Bas Boekestijn, Henricus J. M. Handgraaf, Denise E. Hilling, Jacob Lutjeboer, Jaap Vuijk, Christian H. Martini, Arian R. van Erkel, Rutger W. van der Meer, Fred G. J. Tijl, Frank M. Speetjens, Ellen Kapiteijn, Alexander L. Vahrmeijer

**Affiliations:** 1 Department of Radiology, Leiden University Medical Center, Leiden, The Netherlands; 2 Department of Surgery, Leiden University Medical Center, Leiden, The Netherlands; 3 Biomedical Photonic Imaging Group, University of Twente, Enschede, The Netherlands; 4 Department of Anesthesiology, Leiden University Medical Center, Leiden, The Netherlands; 5 Department of Extra Corporal Circulation, Leiden University Medical Center, Leiden, The Netherlands; 6 Department of Medical Oncology, Leiden University Medical Center, Leiden, The Netherlands

## Abstract

**Background:**

Ocular melanoma is the most common primary intraocular malignancy and has a very poor prognosis once liver metastases occur. The aim of this study was to prospectively assess the efficacy and safety of percutaneous hepatic perfusion with melphalan (M-PHP) using the new second-generation (GEN 2) hemofiltration system in patients with ocular melanoma metastases confined to the liver.

**Methods:**

Prospective, single-center, single-arm, phase II study including patients with unresectable ocular melanoma metastases confined to the liver. Treatment consisted of two M-PHP procedures at 6–8 weeks interval. Procedures were performed using the CHEMOSAT (GEN 2) system with 3 mg/kg melphalan. Primary endpoints were overall response rate (ORR) and best overall response (BOR). Secondary endpoints included overall survival (OS), progression-free survival (PFS), hepatic PFS (hPFS), and safety.

**Results:**

Sixty-four M-PHP procedures were performed in 35 patients between February 2014 and June 2017. The ORR was 72%. BOR was as follows: complete response in 3%, partial response in 69%, stable disease in 13%, and progressive disease in 16%. There was no treatment-related mortality. Fourteen serious adverse events occurred. At a median follow-up of 19.1 months (range 5.6–69.5), median OS was 19.1 months and was significantly longer in responders than in nonresponders (27.5 vs. 11.9 months, p < 0.001). The 1- and 2-year OS was 77% and 43%, respectively. PFS and hPFS were 7.6 and 11.2 months, respectively.

**Conclusions:**

M-PHP using the GEN 2 filter can achieve a high ORR and prolonged survival in patients with liver-only ocular melanoma metastases.

Ocular melanoma is the most common primary intraocular malignancy in adults.^1^ It most frequently arises from melanocytes in the uveal tract, which is subdivided in an anterior part containing the iris (~ 5%) and a posterior part containing the choroid and ciliary corpus (~ 80%).^1^,^2^ The rest of ocular melanomas develop in the conjunctiva (~ 5%) or elsewhere in the orbit (~ 10%). The incidence of uveal melanoma in Europe varies with latitude, being higher in Northern (≥ 8 per million) than Southern Europe (< 2 per million), due to a positive association with Caucasian ethnicity, fair skin, and light eye colour.^4^ Most patients are diagnosed after age 50 years, with a peak range of 65–75 years.^1^^–^5 Despite successful treatment of the primary tumor, up to 50% of patients will eventually develop metastatic disease with predominant liver involvement.^1^^–^3

Metastatic ocular melanoma carries a poor prognosis, because there are no effective systemic treatments. Reported median overall survival (OS) following systemic treatment, including immunotherapy and kinase inhibitors, ranges from 4.4 to 12.7 months with a 1-year OS rate ranging from 29 to 53%.^6^,^7^ Meta-analyses have demonstrated that patients treated with liver-directed therapies had a significantly longer progression-free survival (PFS) and OS compared with patients receiving systemic therapy.^6^,^7^ Liver-directed therapies used to treat ocular melanoma liver metastases include chemoembolization, immunoembolization, radioembolization, isolated hepatic perfusion (IHP), and percutaneous hepatic perfusion with melphalan (M-PHP) (Table 1).^8^^–^30

**Table 1:** Summary of progression-free survival and overall survival following chemoembolization, immunoembolization, radioembolization, isolated hepatic perfusion, and percutaneous hepatic perfusion

First author (year)	Study design	No. of pts	Transarterial catheter-directed therapy and drug	Median PFS (mo)	Median OS (mo)
Agarwala (2004)^8^	Phase I/II, dose-esc.	19	Chemoembolization (cisplatin)	N/A	8.5
Patel (2005)^9^	Phase II	30	Chemoembolization (BCNU)	N/A	5.2
Vogl (2007)^10^	PS, pilot	12	Chemoembolization (mitomycin C)	N/A	21
Schuster (2010)^11^	RS	25	Chemoembolization (fotemustine/cisplatin)	3	6
Gupta (2010)^12^	RS	125	Chemoembolization (mostly cisplatin^a)^	3.8	6.7
Huppert (2010)^13^	PS, pilot	14	Chemoembolization (cisplatin/carboplatin)	8.5	11.5
Edelhauser (2012)^14^	RS	21	Chemoembolization (fotemustine)	7.3	28.7
Valpione (2015)^15^	RS	58	Chemoembolization (irinotecan)	N/A	16.5
Shibayama (2017)^16^	RS	29	Chemoembolization (cisplatin)	6	23
Yamamoto (2009)^17^	RS	53	Immunoembolization vs. chemoembolization (BCNU)	12.4 vs. 4.8	20.4 vs. 9.8
Valsecchi (2015)^18^	Phase II	52	Immunoembolization vs. bland embolization	3.9 vs. 5.9	21.5 vs. 17.2
Gonsalves (2011)^19^	RS	32	Radioembolization (Y-90)	4.7	10
Klingenstein (2013)^20^	RS	13	Radioembolization (Y-90)	N/A	7
Eldredge-Hindy (2016)^21^	RS	71	Radioembolization (Y-90)	5.9	12.3
Tulokas (2018)^22^	RS	16	Radioembolization (Y-90)	5.6	13.5
Gonsalves (2019)^23^	PS	24	Radioembolization (Y-90)	8.1	18.5
Alexander (2000)^24^	Phase I/II	22	Isolated hepatic perfusion (melphalan) ± TNF^b^	9^c^	11^d^
Alexander (2003)^25^	Phase II	29	Isolated hepatic perfusion (melphalan)	8	12.1
Noter (2004)^26^	Phase II	8	Isolated hepatic perfusion (melphalan)	6.7	9.9
van Etten (2009)^27^	Phase I/II	8	Isolated hypoxic hepatic perfusion (melphalan)	6	11
Vogl (2017)^28^	RS	18	Percutaneous hepatic perfusion (melphalan)	12.4	9.6
Karydis (2018)^29^	RS	51	Percutaneous hepatic perfusion (melphalan)	8.1	15.3
Artzner (2019)^30^	RS	16	Percutaneous hepatic perfusion (melphalan)	11.1	27.4

*BCNU* 1,3-bis (2-chloroethyl)-1-nitrosourea, *mo* months, *N/A* not available, *OS* overall survival, *PFS* progression-free survival, *PS* prospective, *pts* patients, *RS* retrospective, *TNF* tumor necrosis factor, *Y*-*90* yttrium-90

^a^Cisplatin (*n* = 122), cisplatin + paclitaxel (*n* = 2), cisplatin + doxorubicin + MMC (*n* = 1)

^b^Isolated hepatic perfusion (*n* = 11), isolated hepatic perfusion with TNF (*n* = 11)

^c^14 months for patients without TNF vs 6 months for patients with TNF (*p* = 0.04)

^d^No difference between both groups (*p* = 0.17)

M-PHP is a minimally invasive, repeatable technique in which the liver is isolated from the systemic circulation and subsequently perfused with high-dose chemotherapy. M-PHP is the only liver-directed therapy that has been investigated in a multicenter, randomized, controlled trial (RCT).^31^ A significant improvement in hepatic and overall PFS was demonstrated in patients treated with M-PHP compared with best alternative care, but the median OS after M-PHP was only 10.6 months. Approximately 40% of patients in this study had extrahepatic metastases, and M-PHP may have had a limited effect on their OS. Additionally, 11% of patients in the study had metastases from cutaneous melanoma.

Concerns regarding the safety of M-PHP have been raised as high rates of hematologic toxicity were reported in prior studies.^31^^–^34 To address the issue of hematologic toxicity, a new hemofiltration system with a second-generation detoxification cartridge (GEN 2 filter) was developed. This filter has a higher melphalan extraction rate than the first-generation filters and was shown to reduce hematologic toxicity.^35^,^36^ So far, only retrospective studies have reported on M-PHP using the GEN 2 filter in ocular melanoma patients.^28^^–^30

The purpose of this study was to prospectively investigate the efficacy and safety of M-PHP using the GEN 2 filter in well-selected patients with unresectable metastases from ocular melanoma confined to the liver.

## Methods

This prospective, single-arm, single-center, phase II study was conducted in accordance with the Declaration of Helsinki, approved by the local ethics committee and registered on www.trialregister.nl (NTR4112). All participants provided written informed consent.

### Patients

Eligible patients were those with histologically proven, unresectable ocular melanoma metastases confined to the liver. All patients were discussed at a multidisciplinary meeting before inclusion. Exclusion criteria are listed in Table 2.

**Table 2:** Exclusion criteria

Laboratory test results	Other
APTT > 1.5 × ULN	Age < 18 or > 75 yr
PT > 1.5 × ULN	Extrahepatic disease (on CECT or FDG-PET/CT)
Leukocytes < 3.0 × 10^9^/L	WHO performance status ≥ 2
Thrombocytes < 100 × 10^9^/L	Severe comorbidity precluding general anesthesia
Creatinine clearance < 40 ml/min	Diabetes with nephropathy
AST > 2.5 × ULN	Active infections
ALT > 2.5 × ULN	< 40% healthy liver tissue
Serum bilirubin > 1.5 × ULN	Other liver disease
ALP > 2.5 × ULN	Vascular anatomy impeding M-PHP
LDH > 2 × ULN^a^	Intracranial lesions with propensity to bleed (on CT/MRI)
	Pregnancy

*ALP* alkaline phosphatase, *ALT* alanine aminotransferase, *APTT* activated partial thromboplastin time, *AST* aspartate aminotransferase, *CECT* contrast-enhanced CT of chest and abdomen, *FDG*-*PET/CT* positron emission tomography with integrated noncontrast enhanced CT and 18F-2-fluoro-2-deoxy-d-glucose as radiotracer, *LDH* lactate dehydrogenase, *M*-*PHP* percutaneous hepatic perfusion with melphalan, *PT* prothrombin time, *ULN* upper limit of normal

^a^Included in the protocol during the course of the study

### Study Protocol

Pretreatment angiography was routinely performed approximately 1 week before the first M-PHP to evaluate hepatic arterial vasculature. If deemed necessary, hepatico-enteric shunts (e.g., right gastric and gastroduodenal artery) were embolized to prevent inadvertent leakage of melphalan.

Treatment consisted of two M-PHP procedures with hepatic artery infusion of melphalan 3 mg/kg (maximum dose 220 mg) at 6–8 weeks interval. Patients demonstrating progressive disease (PD) or unacceptable adverse events after the first M-PHP received only one procedure. If grade 3/4 hematologic toxicity occurred after the first procedure, melphalan dose was reduced by 20–25%. Patients routinely received a subcutaneous injection of granulocyte-colony stimulating factor (pegfilgrastim 6 mg) within 72 h after each M-PHP.

Contrast-enhanced CT of chest and abdomen was performed at baseline, 4–8 weeks after each M-PHP, every 3 months in the first year and every 6 months thereafter until PD occurred. MRI of the liver was performed if lesions were not or poorly visible on CT.

Quality of life (QoL) was assessed using the European Organization for Research and Treatment of Cancer Quality of Life Questionnaire version 3.0 (EORTC QLQ-C30 v3.0). Questionnaires were filled out at baseline, 6 weeks after the first and second M-PHP, and 6 months after the first M-PHP.

All adverse events were monitored continuously throughout the entire study and reported according to the Common Terminology Criteria for Adverse Events version 4.03 (CTCAE v4.03).

### Procedure

All M-PHP procedures were performed using the CHEMOSAT (GEN 2) system (Delcath Systems Inc, New York). General anesthesia was performed with continuous monitoring of the central venous and arterial pressure. Access to the right internal jugular vein (IJV, 10-F sheath), right common femoral vein (CFV, 18-F sheath), and left common femoral artery (5-F sheath) was created. Heparin was administered at an initial dose of 300 U/kg and an activated clotting time of ≥ 450 s was maintained throughout the procedure. A 2.4-F or 2.7-F microcatheter was placed into the hepatic artery at the intended location of infusion. A 16-F double-balloon catheter (Isofuse Isolation Aspiration Catheter, Delcath Systems Inc, New York, NY) was placed in the inferior vena cava (IVC) via the right CFV. The cranial and caudal balloons were inflated at the atriocaval junction and infrahepatic IVC, respectively, to prohibit leakage of melphalan into the systemic circulation. The entire dose of melphalan was infused into the proper hepatic artery or split and infused in the right and left hepatic artery. Melphalan-rich blood was aspirated through catheter fenestrations in a segment between the two balloons, pumped through an extracorporeal hemofiltration system and returned to the patient via the sheath in the right IJV. Once all melphalan was administered, filtration was continued for 30 min to allow complete clearance of melphalan from the liver. The anticoagulant effects of heparin were reversed by protamine sulphate 3 mg/kg, the arterial sheath was removed and hemostasis was achieved using a closure device.^37^

### Endpoints

All imaging was reviewed by independent radiologists using the Response Evaluation Criteria in Solid Tumors (RECIST) 1.1 criteria.^38^ Primary endpoints were overall response rate (ORR) and best overall response (BOR) according to RECIST 1.1. Secondary endpoints were best hepatic response according to RECIST 1.1, OS, PFS, hepatic progression-free survival (hPFS), safety, and QoL.

OS was defined as time of first M-PHP until death or censoring. PFS and hPFS were defined as time of first M-PHP until PD, death, or censoring.

### Statistical Analysis

Kaplan–Meier estimations were used to assess OS, PFS, and hPFS. OS data were censored at the date of last follow-up if patients were still alive. The log-rank test was used to compare curves.

Cox regression analyses were performed to determine possible independent predictors for OS. The Wilcoxon signed-rank test was used to compare scores from questionnaires filled in at baseline and after treatment. *P* < 0.05 was considered statistically significant. Analyses were performed using SPSS 23.0 (SPSS Inc., Chicago, IL).

## Results

### Patient Characteristics

A total of 35 patients (16 men; median age 59 years, range 41–71) were prospectively enrolled between February 2014 and June 2017. Baseline demographic and clinical characteristics of all patients are listed in Table 3.

**Table 3:** Baseline characteristics for all 35 patients with liver metastases from ocular melanoma

Parameter	N	Percentage
Gender		
Men	16	46
Women	19	54
Age, yr [median (range)]	59 (41–71)	…
BMI, kg/m^2^ [median (range)]	25 (20–32)	…
Tumor location		
Choroid	19	54
Choroid with ciliary corpus involvement	12	34
Ciliary corpus	4	11
Type of metastases		
Synchronous	4	11
Metachronous	31	89
Mutations in liver metastases		
GNAQ	21	60
GNA11	12	34
No GNAQ/GNA11	2	6
Time between diagnosis primary tumor and liver metastases, months [median (range)]	28 (0–71)	…
Prior therapy for liver metastases		
Systemic therapy^a^	8	23
Regional therapy^b^	4	11
Regional and systemic therapy	2	6
None	21	60
Radiological aspect metastases		
Hypovascular	3	9
Hypervascular	26	74
Mixed	6	17
Total number of metastases ≥ 10	20	57
Diameter of largest metastasis ≥ 3 cm	14	40
LDH level, IU/L [median (range)]	196 (78–657)	…
Elevated LDH level^c^	8	23
Elevated AFP level^d^	7	20

*AFP* alkaline phosphatase, *BMI* body mass index, *GNAQ* guanine nucleotide-binding protein G(q) subunit alpha, *GNA11* guanine nucleotide-binding protein G(Y) subunit alpha-11, *LDH* lactate dehydrogenase, *SD* standard deviation, *ULN* upper limit of normal

^a^Treatment in randomized phase II SUMIT-trial (selumetinib with dacarbazine vs. placebo) or phase I AEB071-study (protein kinase C inhibitor), ipilimumab, or dendritic cell therapy

^b^Radiofrequency ablation and/or metastasectomy

^c^Normal limits 0–247 for men and women

^d^Normal limits 0–115 U/L for men and 0–98 U/L for women

**Fig. 1:**
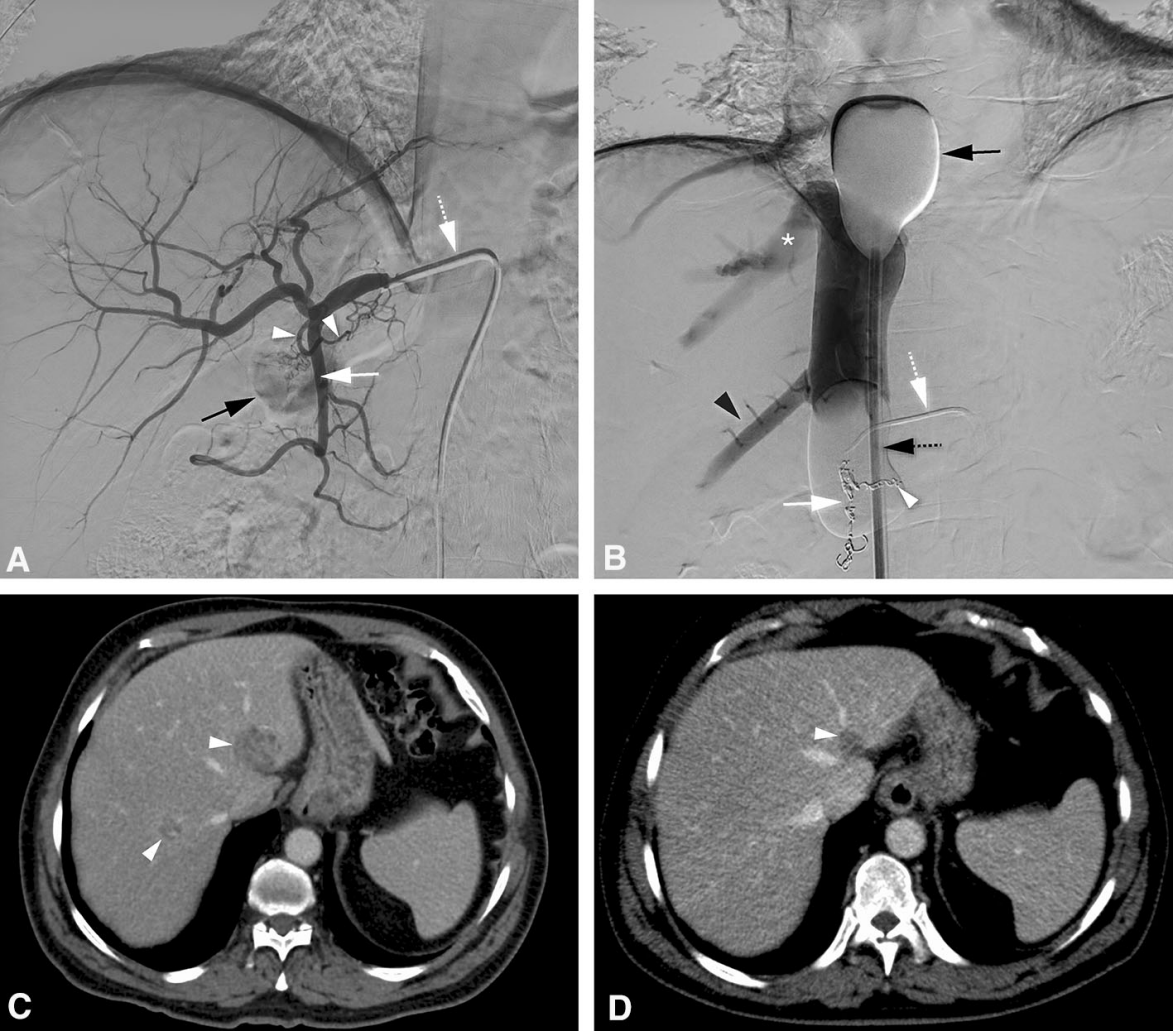
Percutaneous hepatic perfusion with melphalan (M-PHP) in a 66-year-old man with bilobar liver metastases from ocular melanoma. (**a**) Pretreatment angiographic image from the common hepatic artery (CHA) shows a right gastric artery (RGA, white arrowheads) and gastroduodenal artery (GDA, white arrow). Also a 5F macrocatheter in the CHA (dotted white arrow) and the duodenal bulb (black arrow) are shown. The RGA and GDA were successfully coiled. (**b**) Postero-anterior image during venography. The cranial balloon (black arrow) is inflated at the atriocaval junction to prevent flow to the right atrium, and the caudal balloon (dotted black arrow) is inflated in the infrahepatic inferior vena cava (IVC) to prevent retrograde flow to the infrarenal IVC. A 2.7F microcatheter was inserted through the macrocatheter (dotted white arrow) and placed into the proper hepatic artery for the infusion of melphalan. The right hepatic vein (asterisk) and accessory right inferior hepatic vein (black arrowhead) are opacified. Note the coils in the RGA (white arrowhead) and GDA (white arrow). (**c**) Axial CT image in portovenous phase before treatment shows a metastasis in liver segment II and VII/VIII (white arrowheads). A third lesion in segment VI is not shown. (**d**) Axial CT image in portovenous phase after two M-PHP procedures shows reduction in size of the metastasis in liver segment II (white arrowhead). The other two lesions showed a complete radiological response

A total of 64 M-PHP procedures were performed. Twenty-nine of 35 (83%) patients underwent two M-PHP procedures as per protocol. Six of 35 (17%) patients received only one M-PHP due to PD (*n* = 1) or an adverse event (*n* = 5) after the first M-PHP procedure. An example treatment of a study participant is shown in Fig. 1.

### Response Analysis

**Fig. 2:**
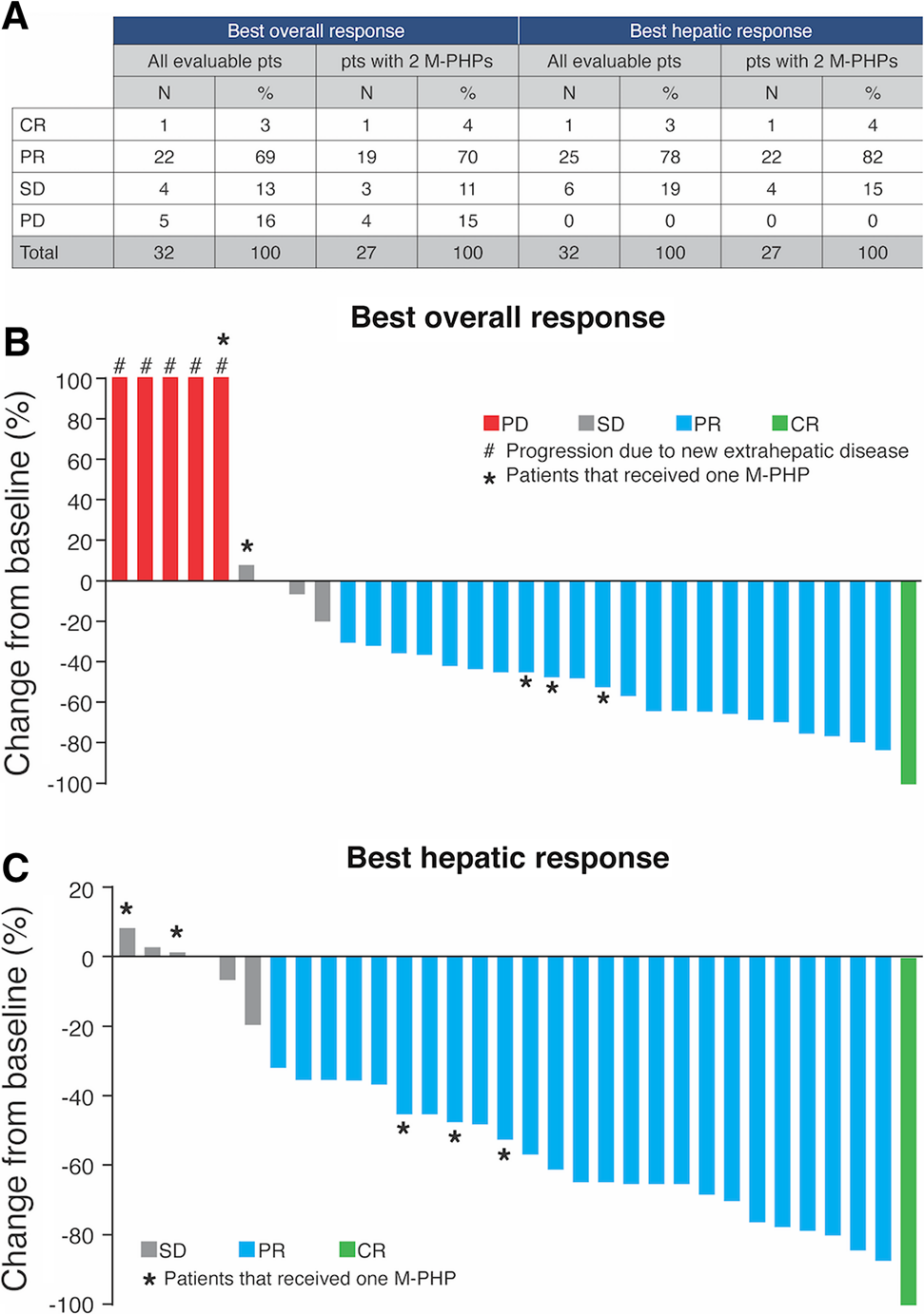
Treatment outcome. (**a**) Best overall response and best hepatic response in all evaluable patients (*n* = 32) and evaluable patients that received two M-PHP procedures (*n* = 27). (**b-c**) Change from baseline in the sum of target lesions at best overall response and best hepatic response in all evaluable patients. *CR* complete response; *M*-*PHP* percutaneous hepatic perfusion with melphalan; *PD* progressive disease; *PR* partial response; *pts* patients; *SD* stable disease

Thirty-two of 35 patients were included in the response analysis (Fig. 2a). In two patients, a therapeutic melphalan dose could not be administered due to peri-procedural complications and therefore no treatment effect could be evaluated. In one patient, target lesions were absent (all lesions with maximal diameter < 1 cm).

The ORR was 72% with complete response (CR) in 3% (*n* = 1) and partial response (PR) in 69% (*n* = 22). A confirmed hepatic response occurred in 26 (81%) patients (3% CR and 78% PR). Five patients had PD as BOR due to extrahepatic metastases; the sum of target lesions in the liver remained stable (*n* = 3) or decreased with > 30% (*n* = 2). The magnitude of BOR and best hepatic response is shown in Fig. 2b, c.

### Survival Analysis

**Fig. 3:**
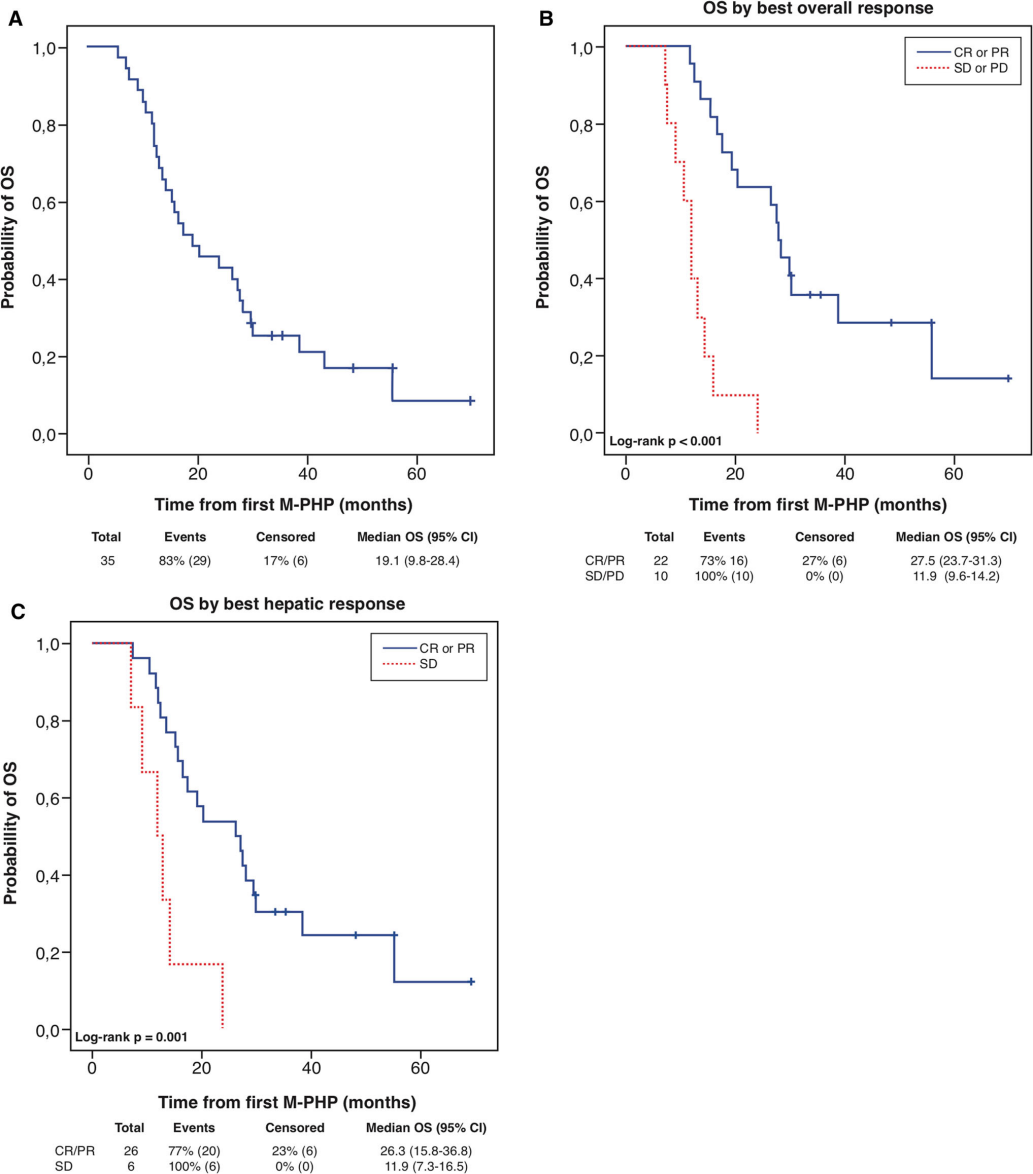
Survival outcomes. (**a**) Kaplan–Meier estimate of OS for all included patients (*n* = 35). (**b-c**) Kaplan–Meier estimates of OS in all evaluable patients stratified by best overall response and best hepatic response. *CI* confidence interval; *CR* complete response; *M*-*PHP* percutaneous hepatic perfusion with melphalan; *OS* overall survival; *PD* progressive disease; *PR* partial response; *SD* stable disease

There was no loss to follow-up. After a median follow-up of 19.1 months, 6 of 35 (17%) patients were still alive. The 1- and 2-year OS was 77% and 43%, respectively. Median OS was 19.1 months for all included patients (*n* = 35; Fig. 3a). Median OS was significantly longer in patients with CR/PR as BOR than in patients with SD/PD as BOR (*p* < 0.001; Fig. 3b). Median OS for patients with CR/PR, SD, and PD as BOR was 27.5 months (95% confidence interval [CI]: 23.7–31.3), 14.2 months (95% CI: 11.4–17.0), and 9.1 months (95% CI: 5.5–12.8), respectively. Median OS also was significantly longer (*p* = 0.001) in patients with CR/PR as best hepatic response than in patients with SD as best hepatic response: 26.3 months (95% CI: 15.8–36.8) versus 11.9 months (95% CI: 7.3–16.5) (Fig. 3c).

Univariate analysis revealed that the presence of a liver metastasis with diameter ≥ 3 cm (*p* = 0.01) and an elevated baseline lactate dehydrogenase (LDH) (> 248 U/L, *p* = 0.03) were significantly associated with a poorer OS. Age (< 65 versus ≥ 65 years, *p* = 0.51), gender (*p* = 0.42), previous local/systemic therapy of liver metastases (*p* = 0.36), mutation status (GNAQ versus GNA11, *p* = 0.57), high tumor burden (> 10 metastases, *p* = 0.65), radiological aspect of metastases (mixed/hypovascular versus hypervascular, *p* = 0.77), and elevated baseline alkaline phosphatase (ALP) (> 115 U/L for men and > 98 U/L for women, *p* = 0.12) were not found to be predictors for OS.

Median PFS was 7.6 months (95% CI: 4.9–10.3) with a 1-year PFS of 26.5%. PFS for patients with a hepatic response was significantly (*p* = 0.001) longer than for nonresponders: 9.3 months (95% CI: 8.6–10.0) versus 5.6 months (95% CI: 2.7–8.5). Median hPFS was 11.2 months (95% CI: 9.0–13.4) with a 1-year hPFS of 35.3%. Median OS in patients with a relatively long hPFS (i.e., ≥ median hPFS of 11.2 months) was significantly (*p* < 0.001) longer than in patients with a relatively short hPFS (< 11.2 months): 29.9 months (95% CI: 11.1–48.7) versus 14.2 months (95% CI: 10.1–183).

Twenty of 34 (59%) patients who eventually showed PD during the course of this study received one or more subsequent treatments (Table 4). Twenty-six of 35 (74%) patients developed extrahepatic metastases during follow-up.

**Table 4:** All patients that received subsequent treatment(s) after showing progressive disease (*n* = 20)

Pt study no.	Progression sites*	Subsequent treatments
1	**Liver**	2x M-PHP, RFA liver
3	**Liver**, bone	RFA liver + ipilimumab^a^
4	**Liver**, bone, lung	2x M-PHP, RTx bone, pembrolizumab, PKC-inhibitor^b^, dacarbazine
5	**Bone**, liver	Ipilimumab
6	**Lung**	Ipilimumab
8	**(Sub)cutis**, parotid gland, rectosigmoid	Resection cutaneous nodes
9	**Liver, subcutis**, lung	RFA liver, resection subcutaneous node
10	**Liver**, muscles, subcutis, retroperitoneum, lymph nodes	RFA liver, RT lymph nodes
11	**Bone**, liver, subcutis	RFA bone and liver
14	**Liver**	Pembrolizumab, PKC inhibitor^b^
16	**Liver, lung**, kidney	PKC-inhibitor^b^
18	**Bone**, liver	PKC-inhibitor^b^
20	**Liver**, peritoneum, retroperitoneum, lung	1x M-PHP, PKC-inhibitor^b^
22	**Liver, subcutis, peritoneum**	Radioembolization, PKC-inhibitor^b^, panitimumab^c^
26	**Liver**, brain	Resection liver metastases
27	**Liver**	2x M-PHP
29	**Liver**, bone	2x M-PHP, RFA liver
30	**Liver**	3x M-PHP
34	**Liver**	PKC-inhibitor^b^
35	**Liver**	RFA liver

*M*-*PHP* percutaneous hepatic perfusion with melphalan, *no.* number, *PKC*-*inhibitor* protein kinase C-inhibitor, *Pt* patient, *RFA* radiofrequency ablation, *RTx* radiation therapy

*Progression sites given in bold represent the initial progression sites

^a^SECIRA-UM study (EudraCT Number: 2011-004200-38)

^b^Phase I study with a protein kinase C-inhibitor

^c^Phase II study with various targeted anti-cancer drugs

### Safety

No deaths occurred. A total of 14 severe adverse events were recorded, including 5 cases of prolonged hospital stay (4–5 days instead of 3 days) and 8 readmissions with a median hospital stay of 6 days (range 1–15). The majority of patients developed grade 3/4 hematologic events with leukopenia (75.6%) and lymphocytopenia (84.8%) being most common. Fourteen grade 3 nonhematologic events occurred, including one case of peri-procedural transient cardiac ischemia, which was managed conservatively and resolved without sequelae. The only patient with a grade 4 nonhematologic event developed a sepsis with bacterial pharyngitis and retropharyngeal abscess formation. This was successfully treated with the intravenous administration of antibiotics and immunoglobulins, followed by percutaneous abscess aspiration. A more detailed description of safety and toxicity has been reported previously as medical authorities and patient organisations requested for the safety profile of M-PHP using the GEN 2 filter to become publicly available at the earliest possible stage.^36^ At that time, the follow-up period was too short to publish data on efficacy.

### Quality of Life

At baseline, 18 of 35 (51%) patients completed the EORTC QLQ-C30 v3.0 form. Return rates of the questionnaire at 6 weeks after the first M-PHP procedure, 6 weeks after the second M-PHP procedure, and 6 months after the first M-PHP procedure were 74% (26/35), 59% (17/29), and 49% (17/35), respectively. Questionnaire scores after treatment did not significantly differ from scores prior to treatment, except for physical functioning which was significantly impaired 6 weeks after the second M-PHP (*p* = 0.011). The level of physical functioning was restored to normal 3 months later (Table 5).

**Table 5:** Quality of life. Scores for each scale evaluated in the EORTC QLQ-C30 v3.0 questionnaire

	Prior to treatment	6 wk after 1^st^ M-PHP	6 wk after 2^nd^ M-PHP	6 mo after 1^st^ M-PHP
	Median (range)	Median (range)	Median (range)	Median (range)
Functional scales (0–100)				
Physical functioning	97 (20–100)	93 (33–100)	87 (33–100)^a^	93 (0–100)
Role functioning	92 (33–100)	67 (17–100)	83 (33–100)	100 (0–100)
Emotional functioning	88 (33–100)	92 (42–100)	83 (58–100)	83 (50–100)
Cognitive functioning	100 (67–100)	100 (50–100)	100 (67–100)	100 (0–100)
Social functioning	100 (50–100)	83 (33–100)	100 (33–100)	100 (50–100)
Symptom scales (0–100)				
Fatigue	6 (0–78)	22 (0–100)	22 (0–78)	11 (0–100)
Nausea and vomiting	0 (0–83)	0 (0–83)	0 (0–33)	0 (0–33)
Pain	0 (0–67)	0 (0–67)	0 (0–50)	0 (0–100)
Dyspnoea	0 (0–67)	0 (0–67)	0 (0–67)	0 (0–33)
Insomnia	0 (0–67)	0 (0–67)	0 (0–100)	0 (0–100)
Appetite loss	0 (0–67)	0 (0–67)	0 (0–67)	0 (0–67)
Constipation	0 (0–33)	0 (0–33)	0 (0–0)	0 (0–67)
Diarrhoea	0 (0–33)	0 (0–67)	0 (0–33)	0 (0–0)
Financial difficulties	0 (0–33)	0 (0–67)	0 (0–67)	0 (0–100)
Global health status/QoL (0–100)				
Global health status/QoL	83 (33–100)	83 (33–100)	83 (42–100)	83 (25–100)

*EORTC QLQ*-*C30 v3.0* European organization for research and treatment of cancer quality of life questionnaire version 3.0, *mo* months, *M*-*PHP* percutaneous hepatic perfusion with melphalan, *QoL* quality of life, *wk* week

^a^Statistically different compared to baseline score, *p* = 0.011. All other scores were not statistically different compared to scores prior to treatment

## Discussion

This study was designed to prospectively investigate the efficacy of M-PHP with the GEN 2 filter in patients with unresectable ocular melanoma metastases confined to the liver. The ORR of 72% and survival rate (median OS 19.1 months; 1- and 2-year OS of 77% and 43%, respectively) appeared to be much longer compared to published data on other treatment modalities and provide convincing evidence for the efficacy of M-PHP.

The prognosis of patients with metastatic ocular melanoma is very poor, and there is a lack of effective systemic therapies. A meta-analysis that included 29 prospective trials that reported patients with metastatic ocular melanoma who were treated with immunotherapy, kinase inhibitors, chemotherapy, or liver-directed therapy, reported a median OS of 10.2 months, 1-year OS of 43%, and median PFS of 3.3 months.^6^ Another recent meta-analysis, which included 78 peer-reviewed articles, reported similar outcomes in patients with metastatic ocular melanoma receiving either surgical, interventional radiology, or systemic treatment.^7^ Median OS across all treatment modalities was 1.07 years and 1-year OS was 52%. In both meta-analyses, patients treated with liver-directed therapies had a significantly longer OS but given the paucity of RCTs the evidence is not compelling. Many studies included in the meta-analyses were retrospective cohort studies with a small sample size and differences in OS between various therapies therefore may be attributable to lead-time, selection, and publication bias.

M-PHP is the only liver-directed therapy for which efficacy was shown in an RCT by Hughes et al. 31 This trial included 93 patients with unresectable liver metastases from either ocular (*n* = 83) or cutaneous (*n* = 10) melanoma. Patients were randomized to M-PHP (*n* = 44) or best alternative care (BAC) (*n* = 49). Approximately 82% of patients in the BAC group received active treatment such as systemic chemotherapy, chemoembolization, radioembolization, and surgery. A significant improvement in hepatic and overall PFS was demonstrated in patients treated with M-PHP: 7.0 versus 1.7 months (*p* < 0.0001) and 5.4 versus 1.6 months (*p* < 0.0001), respectively. The gain in PFS did not result in OS benefit though. The failure to demonstrate OS benefit was most likely caused by the substantial number of patients (40%) with extrahepatic metastases, thereby limiting the optimal effect of a liver-directed therapy. Additionally, almost 60% of patients crossed over to the M-PHP group, receiving M-PHP once disease progression occurred.

The median OS of 19.1 months in the current study compares favorably to the median OS reported in the aforementioned systematic reviews and RCT. It is also longer than the median OS of 15.3 months reported in the largest retrospective study on M-PHP in patients (*n* = 51) with metastatic ocular melanoma.^29^ This study included patients with extrahepatic metastases if these were nonprogressive following previous treatments or amenable to ablative treatment modalities.

Clearly, our favorable survival outcomes can (partly) be attributed to the exclusion of patients with extrahepatic disease. Additionally, we excluded patients with elevated LDH levels (> 2 × ULN) at baseline, and it has been demonstrated that an elevated LDH is associated with a poor OS in patients with metastatic ocular melanoma.^6^,^39^,^40^ Median baseline LDH level was 196 IU/L in our study versus a mean baseline LDH of 524 IU/L in the RCT by Hughes et al.^31^

The hepatic response rate in our study (81%) is much higher than in the study by Hughes et al. (36%) and Karydis et al. (49%).^29^,^31^ The median number of M-PHP procedures that patients received under study protocol was comparable between all these three studies.

The majority of patients received some form of subsequent treatment (i.e., liver-directed therapy and/or systemic therapy) after showing PD. Although this might have influenced survival, all of these therapies were also available and used at the time of the retrospective studies by Karydis et al. (median OS 15.3 months).^29^ This does not apply for the RCT by Hughes et al., which was conducted before checkpoint- and kinase inhibitors were used for metastatic ocular melanoma.^31^ It is unlikely though that subsequent systemic therapies had a large impact on OS as the efficacy of systemic treatments has been limited so far.^3^,^41^^–^44

We found that the median OS in patients with a relatively long hPFS (≥ median hPFS) was significantly longer than in patients with a shorter hPFS (< median hPFS). This, together with the finding that the median OS was significantly longer in responders than nonresponders, suggests that controlling liver disease with M-PHP in patients with liver-only disease improves OS. Ideally, this should be confirmed in a phase III RCT with OS as primary endpoint and no permission for crossover. This, however, has already been proven to be difficult as the FOCUS trial (M-PHP versus best available care, NCT02678572) was recently modified into a single-arm study due to a slow inclusion rate.

We found the presence of a liver metastasis with diameter ≥ 3 cm and elevated LDH level to be poor prognostic factors for OS, as was already reported by Khoja et al. 6 We were unable to confirm their findings that an age ≥ 65 years, male sex, and elevated ALP are also poor prognostic factors for OS.

Concerns have been raised about the safety of M-PHP as prior studies reported high rates of hematologic toxicity. In previous publications, it was demonstrated that the GEN 2 filter has an improved filter extraction rate and improved safety profile.^34^,^35^ We now also provide evidence that M-PHP is well-tolerated with maintenance of QoL. The QoL was only mildly affected with a temporary impaired physical functioning at 6 weeks after the second M-PHP.

The majority of patients (74%) developed extrahepatic metastatic disease during follow-up. These may have been new metastases that developed after M-PHP or metastases that were radiologically occult at baseline. This indicates that many patients with ocular melanoma will suffer from systemic spread for which liver-directed therapy is only a temporarily treatment solution. We recently started a phase I/II study investigating combination therapy of M-PHP with ipilimumab/nivolumab in order to better control both hepatic and extrahepatic disease (CHOPIN trial, NCT04283890). Results of trials investigating the efficacy of check-point inhibitors alone have been disappointing in patients with ocular melanoma metastases. Ocular melanoma cancer cells carry a low tumor mutational burden, which is thought to decrease the likelihood of neoantigen presentation necessary to evoke antitumoral response by T-cells.^45^ Tumor lysis and necrosis induced by M-PHP could potentially provoke antigen release that may stimulate cancer-specific immune response and increase the efficacy of check-point inhibitors.

Our study had several limitations. First, this was a single-arm study with a relatively small sample size. Second, we studied a selected group of patients by applying multiple specific exclusion criteria such as the presence of extrahepatic disease, elevated LDH level, and patient age. The relatively high median OS could therefore partly be attributed to selection.

## Conclusions

Although this prospective study was not designed for direct comparison, the results indicate that M-PHP using the GEN 2 filter is more effective in treating liver metastases from ocular melanoma than systemic therapies. We found a high ORR and median OS of 19.1 months in patients with liver-only ocular melanoma metastases. As responders demonstrated an improved survival compared with nonresponders, controlling liver disease with M-PHP seems to prolong the life expectancy of these patients. Future research should aim to reproduce these results in a multicenter trial with larger study populations and to develop standardized criteria for patient selection.

## References

[1] Jovanovic P, Mihajlovic M, Djordjevic-Jocic J, Vlajkovic S, Cekic S, Stefanovic V (2013). Review article ocular melanoma: an overview of the current status. Int J Clin Exp Pathol..

[2] Isager P, Engholm G, Overgaard J, Storm H (2006). Uveal and conjunctival malignant melanoma in Denmark 1943–97: observed and relative survival of patients followed through 2002. Ophthalmic Epidemiol..

[3] Carvajal RD, Schwartz GK, Tezel T, Marr B, Francis JH, Nathan PD (2017). Metastatic disease from uveal melanoma: treatment options and future prospects. Br J Ophthalmol..

[4] Virgili G, Gatta G, Ciccolallo L (2007). Incidence of uveal melanoma in Europe. Ophthalmology..

[5] Netherlands Comprehensive Cancer Organisation 2011–2018. Available at: https://www.cijfersoverkanker.nl/selecties/dataset_1/img5d1cbba8c92b0. Accessed 3 July 2019.

[6] Khoja L, Atenafu EG, Suciu S (2019). Meta-Analysis in metastatic uveal melanoma to determine progression-free and overall survival benchmarks: an International Rare Cancers Initiative (IRCI) ocular melanoma study. Ann Oncol..

[7] Rantala ES, Hernberg M, Kivelä TT (2019). Overall survival after treatment for metastatic uveal melanoma: a systematic review and meta-analysis. Melanoma Res..

[8] Agarwala SS, Panikkar R, Kirkwood JM (2004). Phase I/II randomized trial of intrahepatic arterial infusion chemotherapy with cisplatin and chemoembolization with cisplatin and polyvinyl sponge in patients with ocular melanoma metastatic to the liver. Melanoma Res..

[9] Patel K, Sullivan K, Berd D (2005). Chemoembolization of the hepatic artery with BCNU for metastatic uveal melanoma: results of a phase II study. Melanoma Res..

[10] Vogl T, Eichler K, Zangos S (2007). Preliminary experience with transarterial chemoembolization (TACE) in liver metastases of uveal malignant melanoma: local tumor control and survival. J Cancer Res Clin Oncol..

[11] Schuster R, Lindner M, Wacker F (2010). Transarterial chemoembolization of liver metastases from uveal melanoma after failure of systemic therapy: toxicity and outcome. Melanoma Res..

[12] Gupta S, Bedikian AY, Ahrar J (2010). Hepatic artery chemoembolization in patients with ocular melanoma metastatic to the liver: response, survival, and prognostic factors. Am J Clin Oncol..

[13] Huppert PE, Fierlbeck G, Pereira P (2010). Transarterial chemoembolization of liver metastases in patients with uveal melanoma. Eur J Radiol..

[14] Edelhauser G, Schicher N, Berzaczy D (2012). Fotemustine chemoembolization of hepatic metastases from uveal melanoma: a retrospective single-center analysis. Am J Roentgenol..

[15] Valpione S, Aliberti C, Parrozzani R (2015). A retrospective analysis of 141 patients with liver metastases from uveal melanoma: a two-cohort study comparing transarterial chemoembolization with CPT-11 charged microbeads and historical treatments. Melanoma Res..

[16] Shibayama Y, Namikawa K, Sone M (2017). Efficacy and toxicity of transarterial chemoembolization therapy using cisplatin and gelatin sponge in patients with liver metastases from uveal melanoma in an Asian population. Int J Clin Oncol..

[17] Yamamoto A, Chervoneva I, Sullivan KL (2009). High-dose immunoembolization: survival benefit in patients with hepatic metastases from uveal melanoma. Radiology..

[18] Valsecchi ME, Terai M, Eschelman DJ (2015). Double-blinded, randomized phase II study using embolization with or without granulocyte-macrophage colony-stimulating factor in uveal melanoma with hepatic metastases. J Vasc Interv Radiol..

[19] Gonsalves CF, Eschelman DJ, Sullivan KL, Anne PR, Doyle L, Sato T (2011). Radioembolization as salvage therapy for hepatic metastasis of uveal melanoma: a single-institution experience. Am J Roentgenol..

[20] Klingenstein A, Haug AR, Zech CJ, Schaller UC (2013). Radioembolization as locoregional therapy of hepatic metastases in uveal melanoma patients. Cardiovasc Intervent Radiol..

[21] Eldredge-Hindy H, Ohri N, Anne PR (2016). Yttrium-90 microsphere brachytherapy for liver metastases from uveal melanoma: clinical outcomes and the predictive value of fluorodeoxyglucose positron emission tomography. Am J Clin Oncol..

[22] Tulokas S, Mäenpää H, Peltola E (2018). Selective internal radiation therapy (SIRT) as treatment for hepatic metastases of uveal melanoma: a Finnish nation-wide retrospective experience. Acta Oncol..

[23] Gonsalves CF, Eschelman DJ, Adamo RD (2019). A prospective phase II trial of radioembolization for treatment of uveal melanoma hepatic metastasis. Radiology..

[24] van Etten B, de Wilt JH, Brunstein F, Eggermont AM, Verhoef C (2009). Isolated hypoxic hepatic perfusion with melphalan in patients with irresectable ocular melanoma metastases. Eur J Surg Oncol..

[25] Noter SL, Rothbarth J, Pijl ME, Keunen JE, Hartgrink HH (2004). Isolated hepatic perfusion with high-dose melphalan for the treatment of uveal melanoma metastases confined to the liver. Melanoma Res..

[26] Alexander HR, Libutti SK, Bartlett DL, Puhlmann M, Fraker DL, Bachenheimer LC (2000). A phase I-II study of isolated hepatic perfusion using melphalan with or without tumor necrosis factor for patients with ocular melanoma metastatic to liver. Clin Cancer Res..

[27] Alexander HR, Libutti SK, Pingpank JF, Steinberg SM, Bartlett DL (2003). Hyperthermic isolated hepatic perfusion using melphalan for patients with ocular melanoma metastatic to liver. Clin Cancer Res..

[28] Vogl TJ, Koch SA, Lotz G (2017). Percutaneous isolated hepatic perfusion as a treatment for isolated hepatic metastases of uveal melanoma: patient outcome and safety in a multi-centre study. Cardiovasc Intervent Radiol..

[29] Karydis I, Gangi A, Wheater MJ (2018). Percutaneous hepatic perfusion with melphalan in uveal melanoma: a safe and effective treatment modality in an orphan disease. J Surg Oncol..

[30] Artzner C, Mossakowski O, Hefferman G (2019). Chemosaturation with percutaneous hepatic perfusion of melphalan for liver-dominant metastatic uveal melanoma: a single center experience. Cancer Imaging..

[31] Hughes MS, Zager J, Faries M (2016). Results of a randomized controlled multicenter phase III trial of percutaneous hepatic perfusion compared with best available care for patients with melanoma liver metastases. Ann Surg Oncol..

[32] Pingpank JF, Libutti SK, Chang R (2005). Phase I study of hepatic arterial melphalan infusion and hepatic venous hemofiltration using percutaneously placed catheters in patients with unresectable hepatic malignancies. J Clin Oncol..

[33] Burgmans MC, de Leede EM, Martini CH (2016). Percutaneous isolated hepatic perfusion for the treatment of unresectable liver malignancies. Cardiovasc Intervent Radiol..

[34] Savier E, Azoulay D, Huguet E (2003). Percutaneous isolated hepatic perfusion for chemotherapy: a phase 1 study. Arch Surg..

[35] de Leede EM, Burgmans MC, Meijer TS (2017). Prospective clinical and pharmacological evaluation of the Delcath System’s second-generation (GEN2) hemofiltration system in patients undergoing percutaneous hepatic perfusion with melphalan. Cardiovasc Intervent Radiol..

[36] Meijer TS, Burgmans MC, Fiocco M (2019). Safety of percutaneous hepatic perfusion with melphalan in patients with unresectable liver metastases from ocular melanoma using the Delcath System’s second-generation hemofiltration system: a prospective non-randomized phase II trial. Cardiovasc Intervent Radiol..

[37] de Leede EM, Burgmans MC, Martini CH, et al. Percutaneous Hepatic Perfusion (PHP) with Melphalan as a treatment for unresectable metastases confined to the liver. *J Vis Exp.* 2016; Jul 31;(113). 10.3791/53795 PMC5091707 27501370

[38] Eisenhauer EA, Therasse P, Bogaerts J, et al. New response evaluation criteria in solid tumours: revised RECIST guideline (version 1.1). *Eur J Cancer.* 2009;45:228–47. 10.1016/j.ejca.2008.10.026 19097774

[39] Nicholas MN, Khoja L, Atenafu EG (2018). Prognostic factors for first-line therapy and overall survival of metastatic uveal melanoma: the Princess Margaret Cancer Centre experience. Melanoma Res..

[40] Valpione S, Moser JC, Parrozzani R (2015). Development and external validation of a prognostic nomogram for metastatic uveal melanoma. PLoS One..

[41] Yang J, Manson DK, Marr BP, Carvajal RD (2018). Treatment of uveal melanoma: where are we now?. Ther Adv Med Oncol..

[42] Luke JJ, Callahan MK, Postow MA (2013). Clinical activity of ipilimumab for metastatic uveal melanoma: a retrospective review of the Dana-Farber Cancer Institute, Massachusetts General Hospital, Memorial Sloan-Kettering Cancer Center, and University Hospital of Lausanne experience. Cancer..

[43] Zimmer L, Vaubel J, Mohr P (2015). Phase II DeCOG-study of ipilimumab in pretreated and treatment-naive patients with metastatic uveal melanoma. PLoS One..

[44] Kottschade LA, McWilliams RR, Markovic SN (2016). The use of pembrolizumab for the treatment of metastatic uveal melanoma. Melanoma Res..

[45] Rozeman EA, Prevoo W, Meier MAJ, et al. Phase Ib/II trial testing combined radiofrequency ablation and ipilimumab in uveal melanoma (SECIRA-UM). Melanoma Res. 2020 Jan 21. [Epub ahead of print] 10.1097/CMR.0000000000000653 31895753

